# Utility of the sentinel node concept for detection of lateral pelvic lymph node metastasis in lower rectal cancer

**DOI:** 10.1186/s12885-017-3408-0

**Published:** 2017-06-19

**Authors:** Shigehiro Yanagita, Yoshikazu Uenosono, Takaaki Arigami, Yoshiaki Kita, Shinichiro Mori, Shoji Natsugoe

**Affiliations:** Department of Digestive Surgery, Breast and Thyroid Surgery, Kagoshima University Graduate School of Medical and Dental Sciences, 8-35-1 Sakuragaoka, Kagoshima, 890-8520 Japan

**Keywords:** Lower rectal cancer, Sentinel nodes, Lateral pelvic lymph nodes, Micrometastasis

## Abstract

**Background:**

There are two lymphatic flows in lower rectal cancer; one along the inferior mesenteric artery and another towards the internal iliac artery. The benefit of dissection of lateral pelvic (LP) lymph nodes (LPLN) remains controversial. This study aimed to clarify the possibility of detecting the sentinel node (SN) of the LP region (LPSN) and examine metastasis, including micrometastasis, using a radio isotope (RI) method.

**Methods:**

In total, 62 patients with clinical (c)T1-T4 rectal cancer were enrolled in this study (11, 16 and 35 patients had tumor located in the upper, middle and lower rectal third, respectively). LPSNs were detected using a radio-isotope method in which 99 m technetium-tin colloid was endoscopically injected into the submucosa in patients with cT1, and into the muscularis propria in patients with cT2, cT3 and cT4. All patients underwent curative resection with lymphadenectomy. LPSN metastases were diagnosed by HE staining, immunohistochemical staining using AE1/AE3 as a primary antibody and by RT-PCR using CEA as a marker.

**Results:**

Of the lower rectal (c)T2–4 tumors, 38.4% had lateral pelvic lymphatic flow that was significantly greater than that of cT1 tumors in the upper and middle thirds of the rectum (*p* = 0.0074). HE and immunohistochemical staining did not detect LPSN metastases but RT-PCR detected micrometastasis of three SNs. The remaining half of LPSNs were immunohistochemically re-examined; in all three cases, isolated tumor cells were detected.

**Conclusion:**

The SN concept may be useful for detecting lateral pelvic lymphatic flow and LPSN metastases, including micrometastasis in lower rectal cancer.

## Background

Total mesorectal excision (TME) for the treatment of rectal cancer has resulted in fewer local recurrences and improved long-term survival, and has become a standard surgical treatment [1–3]. On the other hand, a positive lateral lymph node was shown to be the strongest predictor of both survival and local recurrence [4].

There is a great difference between western countries and Japan regarding the concept of metastasis in the lateral pelvic (LP) lymph nodes (LPLN). In western countries, because LPLN metastasis is considered as a systemic disease, the first treatment for advanced lower rectal cancer is chemo-radiation therapy [5, 6]. In Japan the standard procedure for advanced lower rectal cancer is TME with LPLN dissection [4, 7].

In lower rectal cancer, the lymphatic flow is more complicated compared with cancers in other parts of the colorectum. There are two major lymphatic flows; the first flow is from the tumor along the inferior mesenteric artery and the other flow is from the tumor via lymphatic flow through the lateral ligament and then along the internal iliac artery. The incidence of lateral lymph node metastasis was reported as 20.1% among patients whose lower tumor border was located distal to the peritoneal reflection and whose cancer invaded beyond the muscularis propria. After performing LPLN dissection for this indication, it is expected that the risk of intrapelvic recurrence will decrease by 50%, and that 5-year survival will improve by 8 to 9% [4, 7]. However there remain the problems that urinary function and male sexual function may be impaired after LPLN dissection, even if the autonomic nervous system is completely preserved [4, 7–9].

There are also some problems regarding LPLN metastasis. One problem is the clinical or preoperative diagnosis for the detection of LPLN metastasis. The accuracy of diagnosis of LPLN metastasis using CT is around 60%, and, although that of MRI is better, it is still insufficient [10]. This means that patients with pathological metastasis in LPLNs may be missed. Thus, because of the low sensitivity of diagnosis for LPLN metastasis, some patients without LPLN metastasis undergo lymphadenectomy in those regions, and, conversely other patients with LPLN metastasis do not undergo LPLN dissection.

Although T3-T4 tumors are the indication of LPLN dissection in the Japanese guidelines for the treatment of lower rectal cancer [7], because of the low accuracy of preoperative diagnosis for lymph node metastasis, LPLN dissection is controversial, especially in a laparoscopic TME procedure.

Recently, the concept of the sentinel node (SN), which is the first lymph node to receive lymphatic flow from the tumor, has been introduced. SN navigation surgery (SNNS) is performed clinically in breast cancer [11] and the SN concept has been accepted for early stage gastric cancer [12, 13]. The utility of the SN concept in colorectal cancer has also been reported. Saha et al. described that the SN concept is useful for the detection of aberrant lymphatic drainage [14]. Noura et al. reported that the SN concept is useful for detection of the lateral pelvic SN (LPSN) and for the indication of LPLN dissection by the dye method using indocyanine green and a near-infrared camera system [15]. If the SN concept could be applied to rectal cancer, detection of the LPSN would be clinically beneficial for rectal cancer patients.

The aim of this study was to clarify the possibility of detecting LPSN metastasis, including micrometastasis, using the radio isotope (RI) method for detecting SN in gastric cancer [12].

## Methods

### Patients

Sixty two consecutive patients with cT1-T4 were enrolled in this study. The AJCC/UICC TNM classification and Stage groupings of tumors were used in this study. Eleven, 16, and 35 patients had a tumor located in the upper, middle and lower rectal third, respectively. Overt clinical LPLN metastasis was not detected in any patient by preoperative CT examination. Seventeen cases had metastases in the lymph nodes along the inferior mesenteric artery that were detected by preoperative CT examination. All of the patients underwent curative surgery with lymphadenectomy and provided written, informed consent to participate in the study based on a document approved by our institutional ethics committee. The clinicopathological characteristics those patients enrolled in this study are summarized in Table 1.

**Table 1:** Characteristics of patients

Characteristics		No. (%)
Total no. patients		62
Age (yr)	Median (range) 69 (42–85)	
Sex	Female	20 (32.3)
	Male	42 (67.7)
Clinical stage	I	30 (48.4)
	II	14 (22.6)
	III	15 (24.2)
	IV	3 (4.8)
Clinical T category	T1	15 (24.2)
	T2	20 (32.3)
	T3	25 (40.3)
	T4	2 (3.2)
Clinical N category (along IMA and SRA)	N0	45 (72.6)
	N+	17 (27.4)
Tumor location	upper-middle	27 (43.5)
	lower	35 (56.5)
Histopathological grade	G1	30 (48.4)
	G2	31 (50.0)
	G3	1 (1.6)

### Identification of LPSNs

In this study, lymph nodes that contained the RI tracer and were located along the inferior mesenteric artery were taken as hot nodes (HNs) including patients with nodal metastases along that artery. HNs along the internal iliac artery were defined as LPSNs.

**Fig. 1:**
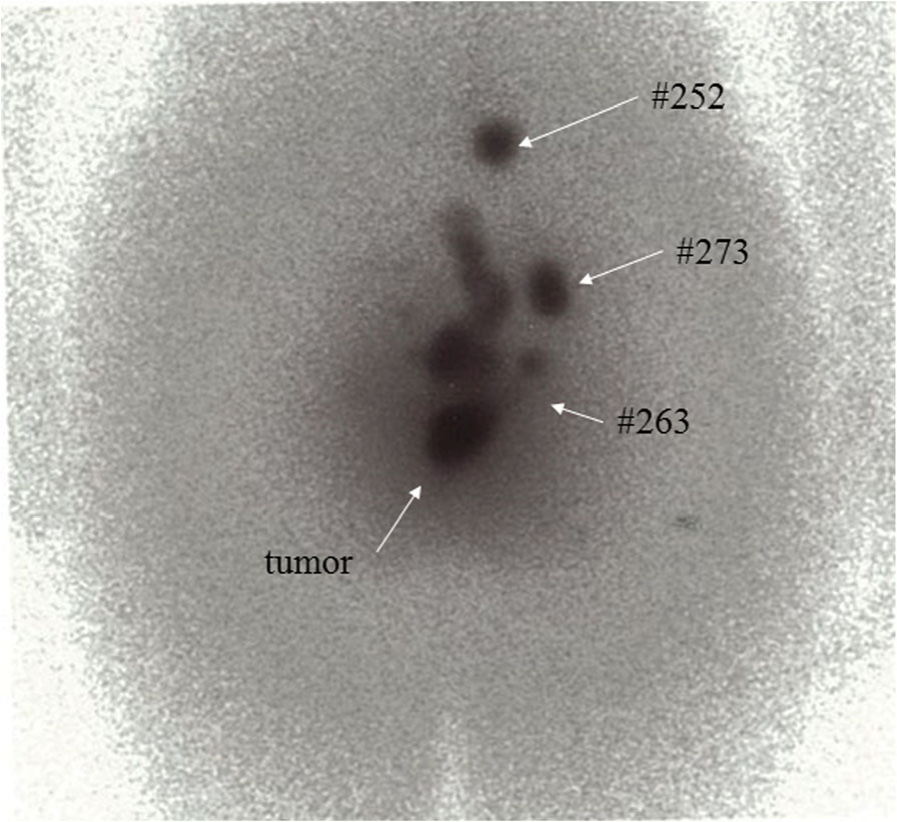
Preoperative lymphoscintigraphy after endoscopic injection of radio isotope. HNs in lateral pelvic lymph nodes are indicated as allows

HNs and LPSNs were mapped as described in previous reports of gastric cancer [12, 16, 17]. In brief, 3 mCi (2 mL) of 99^m^ technetium-tin colloid was endoscopically injected into four sites around the tumor. We changed the depth of the radioisotope injection into the rectal wall to trace tumor specific lymphatics. In cases with a cT1 tumor, the tracer was injected into the submucosa, and, in cases with cT2–4 tumors, the tracer was injected into the muscularis propria. If the endoscope could not pass through the cancer because of its stenosis, technetium-tin colloid was injected only into the anal side of the tumor. These procedures were performed 1 day before surgery. After the endoscopic procedure of radioisotope injection, LPSNs were sometimes confirmed by preoperative lymphoscintigraphy (Fig. 1). During surgery, radioisotope uptake in each lymph node was measured by using the Navigator GPS (RMD Instrument LLC, Watertown, MA, USA). All dissected lymph nodes were mapped after surgery and radioisotope uptake was measured once again. Lymph nodes with signals that were 10-fold above background were considered to be HNs or LPSNs.

### Verification of lymph node metastasis by HE staining and immunohistochemistry

All identified HNs and LPSNs were cut into two uniform pieces at the long-axis of the lymph nodes. One piece was used for HE staining and immunohistochemical staining (IHC), the other piece was used for RT-PCR analysis using the LightCycler® system (Roche Diagnostics). All HNs and LPSNs were stained with HE and were immunohistochemically stained using a monoclonal anti-cytokeratin (CK) antibody cocktail (AE1/AE3; Dako Corporation, Carpinteria, CA, USA) as follows. The tissue sections were deparaffinized in xylene, rehydrated with a graded series of ethanol, and then endogenous peroxidase activity was blocked by a 5-min incubation in 3% hydrogen peroxide in methanol. The sections were subsequently immersed in proteinase K (Dako Corporation) to activate the antigen and were incubated with anti-CK monoclonal antibody (diluted 1:200) for 30 min. After two 5-min washes with phosphate-buffered saline, an avidin-biotin complex and immunoperoxidase were applied (ABC method, Vectastain ABC Kit; Vector Laboratories Inc., Burlingame, CA, USA). Cells positive for CK were visualized using diaminobenzidine tetrahydrochloride and the sections were lightly counterstained with hematoxylin. The negative controls consisted of sections processed in the same manner but without the primary antibody. CK-positive normal gastric mucosa and primary tumor specimens were used as positive controls in all testing. Three independent observers (S.Y., Y.U. and T.A.) evaluated all immunohistochemically stained slides.

### Detection of LPSN metastases using real-time RT-PCR

Sixteen cases were prepared for the LightCycler® system according to a previously described method [16]. This assay was performed based on the hybridization probe method. CEA primer and probe were designed based on those described by Gerhard et al. [18].

### Statistical analysis

Statistical analyses were performed using SAS/JMP statistical analysis software. The clinicopathological variables were analyzed by the Pearson Chi-squared tests. Differences were considered to be statistically significant at *p* < 0.05.

## Results

### Patient backgrounds

Clinicopathological findings of the 62 patients enrolled in this study and. The pathological tumor depth was as follows: 19 (30.6%), nine (14.5%) and 34 (54.8%) patients had pathological T1 (pT1), pT2 and pT3–4 tumors, respectively. Pathologically, 25 patients (40.3%) had lymph node metastases along the inferior mesenteric artery (IMA) and the superior rectal artery (SRA). No patient had LPLN metastases based on HE staining. The accuracy rate of the diagnosis of tumor depth was 93% and 89% in cT1 and cT2–4 respectively. There were no significant correlations between tumor location and the clinicopathological factors. There is no adverse events and morbidities beyond Grade II of Clavien-Dindo classification associated with patients from receiving lymphadenectomy.

### Detection and distribution of HNs and LPSNs in patients with rectal cancer

HNs or LPSNs were detected in 58 cases (detection rate of HNs or LPSNs: 93.5%). Forty five of these 58 cases (77.6%) had HNs only, 12 cases (20.7%) had both HNs and LPSNs and one case (1.7%) had LPSNs only. Regarding the cases with HNs or LPSNs Table 2 shows the details of clinical, pathological information and the distribution of HNs and LPSNs.

**Table 2:** Details of the distribution about the HN’s and LPSN’s location

Case	cT	cN	pT	pN	ly	v	Inferior mesenteric artery	Internal iliac artery	Obturator artery	Common iliac artery	External iliac artery	Inguinal
1	2	0	4	0	+	+						positive
2	2	0	2	0	−	+	positive	positive				
3	2	0	2	1	+	+	positive		positive			
4	3	1	3	0	+	+	positive	positive				
5	3	1	3	1	+	+	positive	positive				
6	4	0	4	0	+	+	positive	positive				
7	3	1	3	0	+	+	positive	positive				
8	2	0	3	0	−	+	positive					positive
9	3	1	3	0	−	+	positive	positive				
10	3	2	2	2	+	+	positive				positive	
11	2	0	1	0	−	−	positive	positive				
12	2	2	3	1	+	+	positive	positive				
13	3	0	2	0	−	−	positive	positive				

The lymphatic flows based on the distribution of HNs and LPSNs were analyzed. Tumors in the lower third of the rectum had significantly greater lateral lymphatic flow compared with tumors located in the middle and upper thirds of the rectum (*p* = 0.0454), and cT2–4 tumors had significantly greater lateral lymphatic flow compared with cT1 tumors (*p* = 0.0039). When the combined tumor location and clinical tumor depth were considered, 37.9% of cT2–4 tumors located in the lower third of the rectum had significantly more lateral lymphatic flows than cT1 tumors located in the upper and middle thirds of the rectum (*p* = 0.0074).

Based on the pathological diagnosis, pT2–4 tumors had significantly more lateral lymphatic flow compared with pT1 tumors (*p* = 0.0235). When the combined tumor location and the pathological tumor depth were considered, 38.5% of pT2–4 tumors located in the lower third of the rectum had significantly more lateral lymphatic flows compared with pT1 tumors located in the upper and middle thirds of the rectum (*p* = 0.0032) (Table 3).

**Table 3:** Correlation of Lateral lymphatic flows in combination of tumor location and tumor depth in patients with rectal cancer

Tumor depth			upper-middle (*n* = 22)		lower (*n* = 36)	*P*-value
clinical	T1	(*n* = 14)	0 (0/7)		0 (0/7)	0.0074
	T2–4	(*n* = 44)	13.3% (2/15)		37.9% (11/29)	
pathological	T1	(*n* = 18)	0 (0/8)		10% (1/10)	0.0032
	T2–4	(*n* = 40)	14.3% (2/14)		38.5% (10/26)	

These data indicated that pT2–4 tumors in the lower third of the rectum had significant tumor-specific lateral lymphatic flows from those tumors.

### LPSNs metastases detected by HE staining and immunohistochemical staining

HE staining and IHC were performed in 58 patients to detect lymph node metastases. HE staining detected LN metastases in the lymph nodes along IMA in 23 patients (39.6%). In 9 of these patients, metastasis was detected in HNs. 14 cases with metastases in non HNs thus the sensitivity of detection of metastases in HNs was 39.1% (9 of 23 patients). LPLN metastases were not detected by HE staining in such patients. There were a total 49 cases without nodal metastases in HNs (14 cases with nodal metastases in non-HNs and 35 cases without nodal metastases by HE staining). In 8 cases of these 49 cases, lymph node metastases were additionally detected by IHC and 6 cases nodal metastases in HNs. LPLN metastases were not detected by IHC. In total the sensitivity for detection of lymph node metastases in HNs was 48.4% (15/31). Regarding LPLN metastasis, neither HE staining nor IHC detected any metastases.

### LPSN metastases detected by RT-PCR

**Fig. 2:**
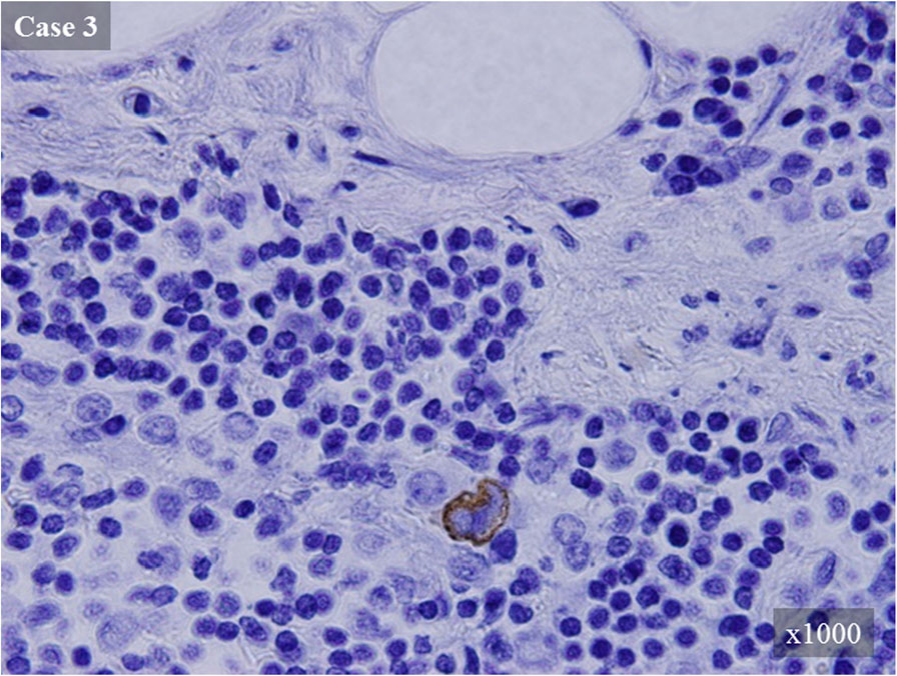
Isolated tumor cells in LPSNs that were detected by immunohistochemical staining. The second half of each LPSN sample was cut into slices 4 μm thick and these slices were immunohistochemically stained using AE1/AE3 as the primary antibody. Representative cases are shown. All LPSN metastases that were detected using RT-PCR were assayed in isolated tumor cells that are indicated with a brown-colored cell membrane

RT-PCR analysis of LPSN metastasis was performed in 16 patients with nodal metastasis. RT-PCR detected LPSN metastases in three patients (Table 4). In these three patients, whole section of the remaining half of the LPSN tissue by 4-μm was performed and was examined using IHC. Isolated tumor cells were detected in all three patients (Fig. 2).

**Table 4:** Cases with LPSN metastases detected by RT-PCR

Case	Tumor location	Gross type	Tumor size (cm)	Histology	pT	pN(IMA^c^)	Number of slides of ITCs/total slides
1	Lower	Depressed	5.5	well^a^	3	1	2/52
2	Middle	Depressed	6.0	mode.^b^	4b	0	1/39
3	Lower	Elevated	3.3	well	3	0	1/480

^a^Well differentiated tubular adenocarcinoma

^b^Moderately differentiated tubular adenocarcinoma

^c^Inferior mesenteric artery

## Discussion

The lymphatic network in the lower rectum is complicated. There are two major lymphatic pathways. One pathway is towards the root of the inferior mesenteric artery via the superior rectal artery and the other pathway is towards the internal iliac artery via the lateral ligament. The lymphatics are anatomically more complicated near the anus, compared with the upper region of the rectum [19]. Additionally, the incidence of LPLN metastases is more frequent in lower rectal cancer than in other tumors in the rectum [20].

In patients with a lower rectal tumor who underwent pelvic side wall dissection, the incidence of pathological LPLN metastases was reported as 4.8%, 7.6% and 15.7% in T1, T2 and T3 tumors, respectively [4]. According to the Japanese Society for Cancer of the Colon and Rectum (JSCCR) Guidelines 2014 for treatment of colorectal cancer, the incidence of pathological LPLN metastases in patients with T2 (MP) and T3 (A) who underwent pelvic side wall dissection was 7.6% and 15.7%, respectively [4], and the indication for LPLN dissection is a T3 tumor [7].

To express these data differently, LPLN dissection is not necessary in 92.4% of T2 tumors and in 84.3% of T3 tumors. This means that accurate diagnosis of LPLN metastasis is important before surgery.

Although metastasis is currently preoperatively examined by various imaging means, the accuracy rate is not sufficient.

In Japan, a randomized controlled study was conducted in patients with clinical stage II and stage III cancer that was located in the lower rectum who underwent mesorectal excision alone or mesorectal excision with LPLN dissection (JCOG0212). The data of postoperative morbidity and mortality have been published [21] and indicated that there was no significant difference in Grade 3–4 postoperative complications such as anastomotic leakage or urinary retention between the two groups. However, that study was conducted based on clinical diagnosis and the pathological diagnosis of LPLN metastasis will not be possible unless TME with lateral pelvic lymph nodes dissection is performed in all cases.

Kobayashi et al. investigated LPLN metastasis using multidetector row computed tomography and reported that its sensitivity and specificity was 78% and 100%, respectively after adaption of a proper cutoff value of 6 mm for the minor axis of a lymph node [22]. Furthermore, Akiyoshi et al. reported that magnetic resonance imaging was useful to determine the indication of LPLN dissection before and after preoperative chemoradiotherapy [23].

The indication of LPLN dissection is a T2–4 tumor [7] and Sugihara et al. discussed that in patients with Stage II tumor with LPLN dissection, the overall survival rate was better than in those without LPLN dissection, because micrometastasis were dissected [4]. At present, there is no preoperative modality to detect lymph node micrometastasis. ‘Micrometastasis’ is important controversial issue at the points of clinical significance and diagnostic method. Based on the morphological or methodological findings ‘micrometastasis’ is also referred to as micrometastasis, occult metastasis, latent metastasis, microinvolvment, and isolated tumor cells (ITC). 6th edition of TNM classification of malignant tumor defined these terms. Micrometastasis was define as no metastasis larger than 0.2 cm and ITC which are usually detected immunohistochemistry (IHC) or molecular methods was defined as individual tumor cells or small cell clusters that do not exceed 0.2 mm in the greatest dimension. Bilchik AJ et al. demonstrated clinical significance of micrometastasis in colon cancer by prospective multicenter trial. All patients with recurrences had SN metastases detected by either HE/IHC or RT-PCR. No patient with no metastases in SNs by HE and RT-PCR has recurred [24]. Based on the results of those investigations, there is possibility that patients with LPLN micrometastasis are targeted for treatment such as surgery or adjuvant chemotherapy. For example it is considerable that cases with histological LPLN marometastases undergo LPLN dissection or cases with LPLN microtmetastases undergo adjuvant chemotherapy. And detection of LPSN metastases may contribute to the efficient decision of those therapy.

The procedure that we used to detect lateral lymphatic flow is tumor specific and, using this procedure, it is possible to detect micrometastasis in LPLN. In the present study, our procedures were not useful for the detection of the SN along the inferior mesenteric artery. However, they were useful for the detection of the SN in the lateral pelvic area in cases that were cN0 for LPLN.

We changed the depth of injection of the radioisotope into the rectal wall according to tumor depth. In T1 tumors the tracer was injected into the submucosa, and in T2–4 tumors, it was injected into the muscularis propria. Regarding lymphatic vessel distribution in the colorectal wall, lymphatic vessels are abundant in the submucosal layer [25]. In small intestine, there are lymphatic network in submucosal and muscular layer [26]. We checked the lymphatic network in rectal wall of several cases by immunohistochemical staining using D2–40 specific for lymphatic vessels as a primary antibody and that network exist in submucosal and muscular layer. Therefore we changed the depth of injection of the radioisotope to trace the tumor specific lymphatic vessel at the invasive front. It is established that there is lymphatic flow from the lower rectal wall to the internal iliac nodes by the lateral ligament [20]. Therefore, based on our data and the histological anatomy, the procedure that we used to detect lymphatic flow from the tumor is tumor-specific. Another problem that is encountered is which side of the LPLN should be dissected by the tumor circumference location. In different words, it is important which sides of LPLNs should be dissected based on the tumor site at the rectum (right, left, anterior, posterior wall). Our procedure may be useful in deciding both the indication of LPLN dissection and which side of the lateral pelvic wall should be dissected. The ability to make such decisions may lead to avoidance of local recurrence after operation.

## Conclusion

The use of 99^m^ technetium-tin colloid may be useful for the detection of tumor specific lateral pelvic lymphatic flow and LPSN metastasis.

## Abbreviations

CK: Cytokeratin
HN: Hot nodes
IHC: Immunohistochemical staining
LP: Lateral pelvic
LPLN: LP lymph nodes
RI: Radio isotope
SN: Sentinel node
SNNS: SN navigation surgery
TME: Total mesorectal excision
